# Impact of dental health on children’s oral health-related quality of life: a cross-sectional study

**DOI:** 10.1186/s12955-015-0283-8

**Published:** 2015-07-07

**Authors:** Aishah Alsumait, Mohamed ElSalhy, Kim Raine, Ken Cor, Rebecca Gokiert, Sabiha Al-Mutawa, Maryam Amin

**Affiliations:** School of Dentistry, Faculty of Medicine and Dentistry, University of Alberta, Edmonton, AB Canada; National School Oral Health Program, Ministry of Health, Kuwait City, Kuwait; School of Public Health, University of Alberta, Edmonton, AB Canada; Faculty of Pharmacy and Pharmaceutical Sciences, University of Alberta, Edmonton, AB Canada; Faculty of Extension, University of Alberta, Edmonton, AB Canada

**Keywords:** Quality of life, Caries, Oral Health, Restorative index, Children, Oral Symptoms

## Abstract

**Background:**

To assess the impact of children’s dental health status (DHS) on their oral health-related quality of life (OHRQoL).

**Methods:**

Participants were 11- and 12-year-old children attending public schools in the Kuwait Capital Region. Children’s DHS was evaluated by clinical examinations and presented using decayed, missed, filled teeth/surface (DMFT/dmft, DMFS/dmfs); restorative (RI), plaque (PI); and pulp, ulcers, fistula, abscess (PUFA) indices. Children’s OHRQoL was assessed using Child’s Perception Questionnaire 11–14 (CPQ_11–14_)_._ Means (SD) and frequencies were used for data description. Different factors were analyzed as predictors of OHRQoL by logistic regression analysis.

**Results:**

A total of 440 children aged 11–12 years (50.7 % females) participated in this cross-sectional study. Mean (SD) DMFT/dmft, RI, PI, and PUFA scores were 2.91(2.75), 0.21 (0.34), 3.59 (1.63), 0.31 (0.85), respectively. The mean total CPQ_11–14_ was 20.72 (16.81). Mean scores of oral-symptoms, functional-limitations, emotional and social well-being were 4.26 (3.32), 5.40 (4.92), 5.48 (6.15), and 5.33 (6.05), respectively. Children with more than four fillings were 95 % less likely to have had oral symptoms than those with no fillings. Children with a DMFT/dmft of 2–3 were 2.8 times more likely to have functional limitation than those with a DMFT/dmft of 0, while children with a DMFT/dmft of more than 4 were 4.4 times more likely to experience limitations. Having two or three non-cavitated lesions reduced the odds of having functional-limitation by 58 %. Children with more than four missing teeth were 45 % more likely to experience emotional stress. Having more than four fissure sealants reduced the odds of having emotional stress by 46 %.

**Conclusions:**

The increase in the number of carious teeth was associated with a limitation in oral functions. Preventive treatment had a positive impact on children’s emotional well-being and restorative treatments improved their oral function.

## Background

Worldwide, dental decay remains one of the most widespread chronic diseases, and oral diseases are the fourth most costly to treat [1]. Oral health is a standard of health of oral and oral-related tissues that contributes to general well-being and enables an individual to eat, speak and socialize without active disease, discomfort or embarrassment [2, 3]. Objective evaluation of oral health status (OHS) includes measures of caries, fluorosis, malocclusion, hypodontia, periodontal diseases and orofacial deformities [4]. Oral health-related quality of life (OHRQoL) measures are subjective indicators based on information provided by individuals about their oral health status and its impact on various aspects of their life [5]. Measures of OHRQoL provide essential information when assessing the treatment needs of individuals and populations, as well as when making clinical decisions and evaluating interventions, services and public health programs [6–9].

Four domains are used to measure OHRQoL: oral symptoms, functional limitations, social well-being and emotional well-being. The domains are interconnected and influence one another as it has been documented in relationships between children’s and adults’ psychological status and functioning [4, 10]. Dental health status (DHS) is, similarly, thought to have a direct impact on overall children’s OHRQoL [11].

The relationships between malocclusion and orofacial deformities (abnormalities in the oral cavity and jaws) and overall OHRQoL, especially in relation to emotional and social well-being domains, are already well-documented [12–14]. Dental caries was also reported to be associated with all components of OHRQoL in a low caries community, where DMFT (decayed, missing, and filled teeth) scores were 1 or less among 12-year-old children [15]. However, in a high caries population, the association was only detected with the oral symptom and functional subscales [16]. Social and emotional well-being subscales were less affected by caries in young children because they attach less importance to their social interactions [12]. Yet, it is not clear which component of the caries, oral hygiene, caries severity indices have an impact on OHRQoL or has a better predictive value in a population with a high level of dental caries.

In this study, we hypothesized that poor DHS, measured by caries/ caries consequences, dental treatments, and hygiene level, is associated with low measures of OHRQoL in all four domains. Therefore, the specific objectives were to: (i) measure DHS by direct examination; (ii) measure OHRQoL by self-administered questionnaire; (iii) evaluate the association between DHS and children’s OHRQoL, and to determine factors that may predict this relationship for school children 11–12 years old.

## Methods

### Study design

This was a cross-sectional study using an OHRQoL survey along with the oral examination of participating children. The study protocol was approved by the University of Alberta Research Ethics Board (Protocol no. 00037434) and the Joint Committee for the Protection of Human Subjects in Research, Kuwait. Informed consent from parents/guardians of every participating child was collected. The study was conducted in accordance with the Helsinki Declaration and was presented following the STROBE guidelines [17].

### Study setting and participants

The study was conducted in the Capital Education/Health Region in Kuwait, where more than 85 % of Kuwaiti children attend public schools, and the research procedures were carried out for 6 months during the 2013 academic year. Participants were 11- and 12-year-old children attending public schools in the Kuwait Capital Region. Seven schools were randomly selected from a list provided by the Ministry of Education Research Department [18]. A letter of information was sent to the selected schools, coupled with an additional information letter to be sent to the parents. All 11–12 children at these schools were approached for participation. The sample size was calculated based on a total of 16,361 students, with a type one error of 0.05, 95 % confidence intervals, and the proportion of 0.5 was estimated to be 375. A 75 % positive response rate was expected; therefore, a total of 500 consents were distributed [19].

### Procedure

A hygienist gave a 5-min presentation to children about the study and sent an information letter along with a consent form home for their parents to sign. Child’s assent was obtained prior to administering the questionnaire and performing the dental examination. Clinical examinations were performed at the Capital public school clinics using fully equipped mobile dental chairs and sterile WHO probes and mirrors.

### Measures

The following measures were used to assess children’s DHS and OHRQoL:

#### Clinical examination

All clinical examinations were conducted at the school clinic using a mobile dental chair, an artificial LED light, and a dental unit. Using the WHO oral health examination criteria, the clinical examinations were conducted by one calibrated examiner. The examiner had training and experience in the use of WHO criteria for the Kuwait National School Oral Health Survey 2013–2014. The examining dentist showed high intra-and inter-examination consistency (kappa = 0.91–0.83). Differences between cavitated and non-cavitated lesions were evaluated according to the International Caries Detection and Assessment System (ICDAS) guideline criteria (https://www.icdas.org). The children’s oral hygiene was evaluated using the Silness-Löe plaque index [20], and the clinical consequences of untreated dental caries were evaluated using the PUFA index [21].

The following indices were recorded as part of the examination: decayed teeth (DT/dt), missing due to decay (MT/mt), filled teeth (FT/ft), DMF teeth (DMFT/dmft), DMF surfaces (DMFS/dmfs), number of sealed teeth, number of non-cavitated teeth, restorative care index (RI) [22], plaque index (PI), and PUFA index for comprehensive oral health examination purposes.

#### CPQ_11–14_ Questionnaire

The Child Perceptions Questionnaire (CPQ_11–14_), developed in Toronto, Canada, by Jokovic et al. [6] and was used to assess each child’s oral impacts on function, life-style activities, general sense of well-being, and relationship with others [11]. The CPQ_11–14_ includes the four domain subscales of oral symptoms (e.g. pain), functional limitations (e.g. difficulty eating/drinking), emotional well-being (e.g. avoiding smiling or laughing around other children), and social well-being (e.g. being asked questions/experiencing comments from other children about his/her mouth). The Arabic version of this questionnaire was translated and validated by Brown and Al-Khayal [16] and provides good psychometric properties (e.g., internal consistency, test-retest reliability). The CPQ_11–14_ instrument can be self-administered or interviewer-administered, with only slight differences in the score results [6]. For this study, we introduced the questionnaire to the children and used the CPQ_11–14_ self-administered form.

The CPQ_11–14_ questionnaire uses Likert-type scales with response options of “Never” = 0, “Once or twice” = 1, “Sometimes” = 2, “Often” = 3, and “Every day or almost every day” = 4 within a recall period of 3 months. Items are grouped into four domains: oral symptoms (6 questions), functional limitations (9 questions), emotional well-being (9 questions), and social well-being (12 questions). Domain and overall OHRQoL scores of the CPQ_11–14_ were calculated by summing all of the responses to items either in the domains or on the whole questionnaire. Lower scores indicated a better OHRQoL.

The questionnaire also contained two global self-rating questions on perceived oral health (with Likert-type scale) responses ranging from “Excellent” = 0, “Very good” = 1, “Good” = 2, “Acceptable” = 3, “Poor” = 4, and one question about the impact of oral health on overall well-being responses ranging from “Not at all” = 0, “Very little” = 1, “Somewhat” = 2, “A lot” = 3 to “Very much” = 4. The latter question was used as a dependent variable in the analysis as a further indicator of OHRQoL.

The Arabic version questionnaire was pre-tested with a group of students, and unclear words were replaced with alternatives that were easier to understand. One hundred and eighteen questionnaires were administered twice, with a two-week gap between attempts; kappa scores for the test/retest questionnaires were 0.87–1.0.

Internal consistency was quantified using Cronbach’s alpha for the CPQ_11–14_ questionnaire as well as each subscale. The intra-class correlation coefficient of repeated questionnaires was used to measure agreement. The item response rate was 100 %, and the results suggested high levels of internal consistency for the overall questionnaire. Reliability, tested by Cronbach’s alpha for the overall CPQ_11–14_ in the sample, was 0.91. The alpha coefficients for emotional and social well-being subscales were 0.83 and 0.81, respectively, which is excellent. The alpha coefficient for the functional limitation subscale (0.7) was acceptable; however, it was only moderate for the oral symptoms subscale (0.58). The intra-class correlation coefficient on repeated applications of the measure was 0.89 (95 % CI = 0.76–0.97), suggesting excellent agreement.

As an index of construct validity, Spearman’s correlation was significant for both global indicators for the total scale (r = 0.23 and 0.335), oral symptoms (r = 0.27 and 0.32), functional limitations (r = 0.184 and 0.32), emotional well-being (r = 0.19 and 0.29) and social well-being (r = 0.14 and 0.22). In addition, all of the constructs of the questionnaire were significantly positively correlated with each other (Table 1).

**Table 1:** Correlations between different components of CPQ_11–14_

	OH self-evaluation	Overall well-being	Oral symptoms	Functional limitations	Emotional well-being	Social well-being	Total CPQ
OH self-evaluation	1						
Degree oral condition affects overall life	0.003	1					
Oral symptoms	0.272^a^	0.324^a^	1				
Functional limitations	0.184^a^	0.316^a^	0.524^a^	1			
Emotional well-being	0.188^a^	0.290^a^	0.474^a^	0.556^a^	1		
Social well-being	0.144^a^	0.222^a^	0.417^a^	0.530^a^	0.615^a^	1	
Total CPQ	0.230^a^	0.355^a^	0.691^a^	0.805^a^	0.862^a^	0.839^a^	1

^a^ Spearman’s correlation is significant at the 0.01 level (2-tailed)

### Data analysis

Data were managed and analyzed using SPSS 21.0 software (IBM Corp., Armonk, N.Y., USA). Data normality was tested using Kolmogorov-Smirnov test. The CPQ_11–14_ responses were used to calculate mean domain scores and overall CPQ_11–14_ scores as well as global self-rating. Indicators of OHRQoL were compared between children grouped by different demographic or DHS variables. Mean differences (i.e., ANOVA) of the DMF and CPQ_11–14_ scores according to different independent variables were evaluated. The correlation between subscales, and global health and oral health questions were evaluated using Spearman’s correlation test.

A new dependent variable was created, to conduct a multivariate logistic regression analysis of OHRQoL, Children were categorized into “negatively affected” (if they recorded the impact on any subscale question as “Often” and/or “Every day or almost every day”) and “not affected”. Oral health status indicators were included as independent variables in multivariate logistic models for OHRQoL together with other controlling socioeconomic indicators, such as gender, number of siblings, mother’s education and mother’s age. Logistic regression models were generated for overall CPQ_11–14_ as well as for every subscale. The level of significance was set at 0.05.

## Results

The response rate was 88 % as 449 children returned with respective parent authorizations. Nine participants were excluded due to the presence of a systemic disorder as reported by parents or child’s uncooperative behavior for the clinical examination or completion of the questionnaire.

The final sample was composed of 440 participants, of which 50.7 % were female. Almost 46.4 % of the children were from families with 2–4 children, while 34 (7.7 %) of them had no siblings. Almost half of the children’s mothers had a college degree and were younger than 40 years of age. The participants’ demographics are summarized in Table 2.

**Table 2:** Basic demographics of participants

Variable	Number (%)
Gender	
Male	217 (49.3)
Female	223 (50.7)
Mother’s education	
High school or less	94 (21.4)
More than high school	106 (24.1)
College	203 (46.4)
Post-college	27 (6.1)
Number of children in the family	
Only child	34 (7.7)
2–4	204 (46.4)
More than 4	202 (45.9)
Mother’s age	
Under 40	227 (53.0)
40 and over	201 (47.0)

### Dental health status

Mean (SD) DT/dt, DMFT/dmft and DMFS/dmfs were 1.96 (2.24), 2.91(2.75), and 5.71 (6.94), respectively. Although 23.9 % of the children had DMFT/dmft = 0. Per child, the mean number of non-cavitated carious teeth was 2.34 (2.17) and mean sealed teeth 1.78 (2.56), while the mean RI was 0.21 (0.34). The mean plaque index was 3.59 (1.63) and the mean PUFA index was 0.31 (0.85). DHS and separate components of DMF/dmf can be reviewed in Table 3.

**Table 3:** Demographics and DHS measures

Variable	DT/dt	MT/mt	FT/ft	DMFT/dmft	DMFS/dmfs	No. of sealed teeth	No. of non-cavitated teeth	RI	Plaque index	PUFA score
Gender										
Male	2.0 (2.2)	0.4 (1.0)	0.7 (1.2)	3.1 (2.8)	6.4 (8.0)^a^	1.9 (2.5)	2.3 (2.2)	0.2 (0.3)	0.9 (0.4)	0.4 (0.9)
Female	1.9 (2.3)	0.2 (0.6)	0.6 (1.2)	2.7 (2.6)	4.9 (5.8)^b^	1.7 (2.6)	2.4 (2.3)	0.2 (0.3)	0.9 (0.4)	0.3 (0.7)
Mother’s education										
High school or less	2.9 (2.8)^a^	0.3 (0.9)	0.6 (.9)^a^	3.8 (3.1)^a^	7.6 (7.9)^a^	1.5 (2.4)	3.0 (2.4)^a^	0.2 (0.3)^a^	1.0 (0.4)	0.5 (1.2)
More than high school	1.7 (2.0)^b^	0.3 (0.8)	0.6 (.9)^a^	2.5 (2.6)^b^	5.3 (6.8)^b^	1.9 (2.6)	2.3 (2.1)^b^	0.2 (0.3)^a^	0.9 (0.4)	0.3 (0.8)
College	1.7 (2.0)^b^	0.3 (0.9)	0.7 (1.2)^a^	2.7 (2.6)^a^	5.0 (6.5)^b^	1.8 (2.6)	2.1 (2.1)^b^	0.2 (0.4)^a^	0.9 (0.4)	0.3 (0.7)
Post-college	1.0 (1.3)^b^	0.1 (0.4)	2.1 (2.4)^b^	3.3 (3.4)^a,b^	4.9 (5.1)^b^	1.7 (1.9)	0.7 (1.1)^b^	0.5 (0.4)^b^	0.8 (0.3)	0.2 (0.5)
Number of children in the family										
Only child	2.7 (3.1)^b^	0.0	0.0	2.7 (3.1)^a^	3.7 (4.7)	0.0	1.3 (0.6)	0.2 (0.3)	0.8 (1.0)^a^	
2–4	1.6 (2.1)^a^	0.3 (0.8)	0. 7 (1.2)	2.5 (2.6)^a^	4.8 (6.4)	1.9 (2.7)	2.4 (2.2)	0.2 (0.4)	0.8 (0.4)^a^	0.3 (0.7)
More than 4	2.7 (2.4)^b^	0.3 (0.9)	0.6 (1.1)	3.2 (2.8)^b^	6.6 (7.5)	1.6 (2.4)	2.4 (2.2)	0.2 (0.3)	1.0 (0.4)^b^	0.4 (1.0)
Mother age										
Under 40	2.0 (2.2)	0.3 (0.8)	0.6 (1.1)	2.9 (2.7)	5.5 (6.55)	1.7 (2.3)	2.5 (2.2)	0.2 (0.3)	0.9 (0.3)	0.3 (0.8)
40 and over	1.9 (2.2)	0.3 (0.9)	0.7 (1.2)	2.9 (2.8)	5.8 (7.41)	1.9 (2.8)	2.2 (2.2)	0.2 (0.4)	0.9 (0.4)	0.3 (0.9)

Within columns, means followed by different superscript letters represent statistical differences among groups by *t*-test or ANOVA (with LSD post-hoc analysis)

D/d = decayed; F/f = filled; M/m = missing; S/s = surface; T = permanent teeth; t = primary teeth; RI = Restorative index; PUFA = Pulp, Ulcer, Fistula, Abscess

Male children had significantly higher DMFS/dmfs than female children (Table 3). Children without siblings and those from families with more than four children had a significantly higher level of dental decay and worse oral hygiene (DT/dt and plaque index) than children from families with 2–4 children. Children from families of more than four children had a significantly higher DMFT/dmft than children from smaller families. Furthermore, those whose mothers had less than a high school education had significantly higher DT/dt, DMFT/dmft, DMFS/dmfs and non-cavitated carious teeth than those whose mothers had a higher education. Children of mothers with a college education had higher FT/ft and RI. There was no significant correlation between mothers’ age and caries, RI, plaque or PUFA indices. The DHS variables according to demographics are summarized in Table 3.

### Oral health-related quality of life

A total of 74.2 % of children reported at least one negative impact on their quality of life by responding with “Often” and/or “Every day or almost every day” in the questionnaire. In the oral symptoms domain, 29.9 % responded to at least one question with “Often” and/or “Every day or almost every day”, while 38.7 % did so in the functional limitations domain, 35.5 % in the emotional well-being domain, and 29.7 % in the social well-being domain.

The mean (SD) total CPQ_11–14_ score was 20.72 (16.81). Mean scores for subscales were 4.26 (3.32) for oral symptoms, 5.40 (4.92) for functional limitations, 5.48 (6.15) for emotional well-being, and 5.33 (6.05) for social well-being. Almost 78 % of the participating children evaluated their oral health as excellent or very good, while only 5 % evaluated it as fair or poor. The mean overall self-evaluation of the effect of OH on their life was 0.69 (0.95), with 82.6 % reporting “not at all or very little” and 5 % reporting that it affects their life “a lot or very much”.

The study’s male children had significantly better emotional well-being than the female children, with no significant differences between genders in overall self-evaluation, total CPQ_11–14_ scores, or the other subscales (Table 4). Children whose mothers had a high school or post-college education had higher total CPQ_11–14_ scores, emotional well-being and social well-being than those whose mothers had lower than high school or post-college education. Children whose mothers were older than 40 years had a better self-evaluation of their OH.

**Table 4:** Demographics and CPQ_11–14_ scores

Variables	Overall OH self-evaluation (0–4)	Degree oral condition affects overall life (0–4)	Total CPQ (0–144)	Oral symptoms (0–24)	Functional limitations (0–36)	Emotional well-being (0–36)	Social well-being (0–48)
Gender							
Male	0.69 (0.98)	0.65 (0.96)	20.45 (16.21)	4.51 (3.52)	5.45 (4.59)	4.90 (5.67)^a^	5.59 (6.44)
Female	0.71 (0.90)	0.71 (0.90)	21.26 (17.74)	4.11 (3.26)	5.46 (5.27)	6.26 (6.79)^b^	5.43 (5.99)
Mother’s education							
High school or less	0.88 (1.05)	0.82 (1.02)	24.33 (18.15)^a^	4.60 (3.86)	5.59 (5.14)	7.35 (6.94)^a^	6.8 (7.35)^a^
More than high school	0.64 (0.90)	0.65 (0.90)	21.54 (17.61)^b^	4.43 (3.40)	5.59 (5.08)	5.82 (6.55)^b^	5.70 (5.82)^b^
College	0.64 (0.88)	0.63 (0.88)	18.44 (15.08)^b^	4.02 (3.05)	5.18 (4.50)	4.55 (5.44)^b^	4.69 (5.55)^b^
Post-college	1.17 (1.47)	1.0 (1.41)	35.0 (30.61)^a^	6.50 (5.47)	9.33 (10.60)	9.83 (10.83)^a^	9.33 (10.86)^a^
Number of children in the family							
Only child	1.50 (0.71)	0.81 (0.63)	35.50 (16.26)	8.00 (4.24)	7.50 (2.12)	7.50 (0.71)	12.50 (9.19)
2–4	0.75 (0.94)	0.74 (0.94)	21.11 (17.28)	4.26 (3.30)	5.69 (5.01)	5.61 (6.41)	5.55 (6.48)
More than 4	0.63 (0.93)	0.61 (0.83)	20.44 (16.70)	4.32 (3.48)	5.15 (4.90)	5.58 (6.24)	5.39 (5.82)
Mother’s age							
Under 40	0.78 (0.97)^a^	0.78 (0.97)^a^	21.43 (17.75)	4.21 (3.41)	5.67(5.13)	5.71 (6.45)	5.84 (6.75)
40 and over	0.60 (0.89)^b^	0.57 (0.86)^b^	20.21 (16.11)	4.41 (3.36)	5.20 (4.73)	5.49 (6.15)	5.12 (5.47)

Within columns, means followed by different superscript letters represent statistical differences among groups by *t*-test or ANOVA (with LSD post-hoc analysis)

D/d = decayed; F/f = filled; M/m = missing; S/s = surface; T = permanent teeth; t = primary teeth; RI = Restorative index; PUFA = Pulp, Ulcer, Fistula, Abscess

### DHS and OHRQoL

Children with DMFT/dmft and DMFS/dmfs of less than 4 and those with less than two carious teeth (DT/dt < 2) had a significantly better self-evaluation of the impact of their dental health on their overall life compared with their counterparts (Table 5). Children with two or more carious teeth suffered from significantly higher oral symptoms and functional limitation compared with those with less than two carious teeth. Children with a PI score of more than two had significantly higher total CPQ_11–14_, oral symptoms and functional limitation scores. The total CPQ_11–14_ scores and subscales are summarized in Table 5.

**Table 5:** DHS and CPQ_11–14_ scores

CPQ components	OH self-evaluation		Overall well-being		Total CPQ		Oral symptoms		Functional limitations		Emotional well-being		Social well-being	
DHS indices	Mean	SD	Mean	SD	Mean	SD	Mean	SD	Mean	SD	Mean	SD	Mean	SD
DMFT/dmft														
0	0.92	0.90	0.52	0.78	20.09	15.57	4.216	3.17	4.80	4.08	5.58	5.79	5.05	5.88
1	0.80	0.93	0.59	0.80	18.26	13.00	3.66	2.80	5.04	4.85	4.92	4.66	4.40	4.61
2 or 3	0.84	0.90	0.63	0.87	20.64	15.15	4.13	3.16	5.56	4.34	5.36	5.34	5.72	6.42
More than 4	0.91	0.95	0.92*	1.13	22.34	20.27	4.68	3.74	5.84	5.90	5.78	7.56	5.59	6.38
DMFS/dmfs														
0	0.92	0.90	0.52a	0.78	20.09	15.57	4.22	3.17	4.80	4.08	5.58	5.79	5.05	5.88
1	0.71	0.90	0.74a	0.86	17.34	11.40	3.55	2.94	5.00	4.69	4.87	4.55	3.47	3.46
2 or 3	0.92	0.98	0.51a	0.85	18.94	13.33	3.79	2.71	5.16	3.99	4.81	4.67	5.51	5.36
More than 4	0.88	0.91	0.83*	1.04	22.17	18.92	4.56	3.61	5.83	5.53	5.77	6.95	5.73	6.64
DT/dt														
0	0.88	0.88	0.58	0.82	19.70	14.83	4.12	3.01	5.07	4.10	5.25	5.76	4.87	5.69
1	0.89	0.94	0.60	0.87	17.70	13.62	3.32	2.60	4.56	4.45	5.14	5.23	4.86	5.19
2 or 3	0.85	0.89	0.82*	1.06	23.43	17.48	4.78*	3.43	6.27*	5.36	6.13	6.33	6.19	6.62
More than 4	0.91	1.02	0.80*	1.04	21.32	20.99	4.64*	4.07	5.48*	5.82	5.31	7.32	5.41	6.55
MT/mt														
0	0.87	0.92	0.67	0.91	20.37	16.46	4.14	3.20	5.22	4.77	5.55	6.14	5.22	5.95
1	1.03	0.85	0.72	1.00	21.85	16.03	5.34	3.37	5.80	4.83	4.69	5.34	5.53	6.29
2 or 3	0.65	0.83	1.00	1.23	24.18	23.24	4.57	4.87	7.43	7.15	5.55	7.22	6.52	7.48
More than 4	1.13	1.36	1.00	1.41	22.63	17.33	4.50	3.25	5.88	3.64	6.13	8.08	6.13	6.13
FT/ft														
0	0.92	0.93	0.61	0.88	21.03	16.33	4.22	3.35	5.35	4.71	5.64	5.87	5.38	5.89
1	0.87	1.00	0.80	0.95	19.72	17.19	4.68	3.68	5.11	4.81	4.73	5.75	5.40	6.54
2 or 3	0.71	0.73	0.96	1.20	21.47	19.52	4.16	2.89	6.42	6.23	6.14	7.69	5.06	6.20
More than 4	0.85	0.81	0.75	1.12	18.94	15.47	3.32	2.14	4.67	4.52	4.79	7.51	5.20	6.26
RI														
0– < 0.2	0.89	0.93	0.73	0.97	21.10	16.93	4.24	3.47	5.55	5.05	5.73	6.14	5.58	5.96
0.2–0.5	0.91	1.04	1.02	1.05	24.91	20.52	5.66*	4.11	6.46	5.83	6.15	6.84	6.68	8.28
0.5 and more	0.76	0.84	0.73	1.04	18.38	16.10	3.45	2.41	5.39	5.19	4.77	6.74	4.77	5.32
Plaque index														
0– < 1	0.92	0.94	0.68	0.97	20.62	15.09	4.27	3.07	5.34	4.57	5.53	5.81	5.17	5.76
1– < 2	0.83	0.88	0.70	0.92	20.57	19.35	4.12	3.66	5.41	5.40	5.39	6.72	5.57	6.51
2–3	0.50	0.84	1.33	1.03	35.25*	14.24	8.00*	2.61	8.40*	6.39	6.40	5.18	6.33	6.83
PUFA score														
0	0.86	0.88	0.68	0.94	20.62	15.52	4.18	3.15	5.39	4.63	5.47	5.78	5.34	5.93
1	1.04	1.15	0.81	0.96	22.40	21.92	4.79	3.64	5.85	5.90	6.30	8.41	5.44	6.57
More than 1	0.87	0.97	0.70	1.02	19.56	23.33	4.43	4.69	4.79	6.52	4.40	6.41	5.07	6.99

Within columns, means followed with different superscript letters represent statistical differences among groups by *t*-test or ANOVA (with LSD post-hoc analysis) (p < 0.05). * indicate statistical significant result

### Predictors of OHRQoL

Factors associated with a negative impact on OHRQoL were identified as predictors of OHRQoL using logistic regression analysis (Table 6). Having adjusted for a potential confounding effect in the logistic regression analysis, it was found that participating children with a DMFT of 2 or 3 were 3.8 times more likely to have their quality of life affected than those with a DMFT of 0 (OR = 3.80, 95 % CI: 1.13–12.87), while having a DMFT of more than 4 increased the odds to 11.5 times (OR = 11.46, 95 % CI = 1.80–73.02). Children with more than 4 carious teeth were two times more likely to be affected in the 3 months preceding the study than those who were caries-free (OR = 2.21, 95 % CI: 1.04–2.01). Having 2 or 3 filled teeth decreased the odds of having an impact on quality of life by 62 %, while having more than 4 fillings reduced it by 81 %. For children with a PUFA score of more than 1, it was 33 % more likely that their life was affected in the 3 months prior to the study (Table 6). The only predictor of oral symptoms was number of filled teeth (Table 7). Children with more than four fillings were 95 % less likely to have had oral symptoms in the previous 3 months than those with no fillings (OR = 0.05, 95 % CI: 0.01–0.59).

**Table 6:** Odds ratio (95 % CI) for negative impact on OHRQoL from multivariate logistic regression analysis final model^a^

Variables	Sig.	Odds ratio	95 % CI	
			Lower limit	Upper limit
DMFT/dmft				
0	-	Reference		
1	0.128	2.758	0.746	10.191
2 or 3	0.032	3.804	1.125	12.867
More than 4	0.010	11.459	1.798	73.021
DT/dt				
0	-	Reference		
1	0.085	0.475	0.204	1.107
2 or 3	0.184	0.480	0.162	1.417
More than 4	0.042	2.205	1.041	3.014
FT/ft				
0	-	Reference		
1	0.076	0.545	0.279	1.065
2 or 3	0.048	0.377	0.143	0.993
More than 4	0.043	0.187	0.032	0.997
PUFA score				
0	-	Reference		
1	0.582	0.812	0.386	1.705
More than 1	0.027	1.327	1.021	2.883

^a^Only significant variables (p < 0.05) were kept in the final model. Odds ratios were adjusted for other variables in the model

D/d = decayed; F/f = filled; M/m = missing; T = permanent teeth; t = primary teeth; PUFA = Pulp, Ulcer, Fistula, Abscess

**Table 7:** Odds ratio (95 % CI) for negative impact on different OHRQoL domains from multivariate logistic regression analysis final model^a^

Variables	Sig.	Odds ratio	95 % CI	
			Lower limit	Upper limit
Oral symptoms				
FT/ft				
0	-	Reference		
1	0.220	0.383	0.083	1.776
2 or 3	0.056	0.169	0.027	1.049
More than 4	0.017	0.053	0.005	0.587
Oral functions				
DMFT/dmft				
0	-	Reference		
1	0.436	1.676	0.457	6.155
2 or 3	0.036	2.785	1.166	8.957
More than 4	0.028	4.428	1.261	25.785
Non-cavitated lesions				
0	-	Reference		
1	0.713	0.864	0.396	1.886
2 or 3	0.018	0.421	0.205	0.864
More than 4	0.072	0.509	0.244	1.063
Mother’s education				
High school or less	-	Reference		
More than high school	0.445	0.764	0.383	1.524
College	0.020	0.471	0.249	0.889
Post-college	0.832	0.789	0.088	7.060
Emotional well-being				
MT/mt				
0	-	Reference		
1	0.346	0.651	0.266	1.591
2 or 3	0.034	1.234	0.061	0.897
More than 4	0.025	1.446	1.072	2.759
Fissure sealants				
0	-	Reference		
1	0.254	1.450	0.766	2.745
2 or 3	0.133	1.649	0.859	3.168
More than 4	0.049	0.538	0.276	0.949
Mother’s education				
High school or less	-	Reference		
More than high school	0.018	0.466	0.248	0.878
College	<0.001	0.286	0.158	0.518
Post-college	0.549	0.597	0.110	3.224
Social well-being				
Mother’s education				
High school or less	-	Reference		
More than high school	0.927	1.029	0.553	1.917
College	0.003	0.398	0.217	0.731
Post-college	0.787	1.259	0.237	6.689

^a^Only significant variables (p < 0.05) were kept in the final model. Odds ratios were adjusted for other variables in the model

D/d = decayed; F/f = filled; M/m = missing; T = permanent teeth; t = primary teeth; PUFA = Pulp, Ulcer, Fistula, Abscess

Children with a DMFT/dmft of 2 or 3 were 2.8 times more likely to have limitations in their oral function than those with a DMFT/dmft of 0, while children with a DMFT/dmft of more than 4 were 4.4 times more likely to experience limitations (Table 7). Having two or three non-cavitated lesions reduced the odds of having functional limitations by 58 %. Children whose mothers had at least one college degree were 53 % less likely to have functional limitations than those whose mothers had only a high school education or less.

Children with two or three missing teeth were 23 % more likely to face emotional stress, while those with more than four missing teeth were 45 % more likely to experience emotional stress. Having more than four fissure sealants reduced the chances of having emotional stress by 46 %. Children whose mothers had a high school or college education were 53 % and 71 % less likely, respectively, to face emotional stress due to their teeth than those whose mothers had less than a high school diploma. None of the studied factors showed an association with OH self-evaluation and/or the overall well-being global rating.

## Discussion

This study was conducted to evaluate the association between DHS and OHRQoL and to determine which components of dental health that may be have an impact on OHRQoL. In the study population, the prevalence of caries experienced by the children (76 %) was very close to the percentage of children experiencing oral impacts (74.2 %) in the 3 months preceding the study. The overall mean of children’s CPQ_11–14_ was generally better than that in a study undertaken previously in the region [16] and very similar to studies undertaken in other countries [13]. The improved OHRQoL may be due to the presence of a school-based program that provides treatment, education, and prevention services in the region.

In the study population, the mean CPQ_11–14_ score was relatively high; however, it was consistent with high DMFT level. Male children had a higher DMFS than female children; they also reported better emotional well-being. A gender difference was observed only in the emotional well-being subscale in our study, and this is consistent with the results reported previously by Foster Page et al. [13]. This observation can perhaps be explained by the assumption that females are more concerned about their health and appearance than males [13] and are also more susceptible to emotional stress than males as noted in previous studies [23].

Mothers’ education has been found as one of the major determinants of children’s OHRQoL in previous reports [24, 25]. In our study, mothers’ education was also one of the major determinants of children’s DH status and RI. However, children of uneducated and well-educated mothers had lower overall OHRQoL and, among the four components, emotional and social well-being subscales were affected the most. This may indicate that mothers’ education level may not be a predictor of the OHRQoL in Kuwaiti children. i.e., children’s of mothers’ with a college education had better OHRQoL than children’s of mothers’ with a post-college education. In addition, children of mothers with less than a high school education had a high number of carious teeth, while children of mothers with a post-college education had a high number of filled teeth. In other words, while the experience of caries was the same in both groups, the level of untreated caries was higher in children with uneducated mothers. Nonetheless, the overall OHRQoL was lower in both groups, which may suggest that both untreated and treated caries can negatively affect children’s emotional and social well-being. One possible reason is because children’s DH status may upset their mothers and mothers, in turn, may transfer this stress to the child emotionally [24–26]. In contrary to a previous study [24] reporting no association between mothers’ age and child’s OHRQoL, in our study, children with older mothers, had a better self-evaluation of their OHS and overall well-being.

Similar to previous reports [7, 13, 14, 16, 27–29], the results of our study also suggest that OHS is associated with children’s OHRQoL. While DMFT, DMFS and DT scores greater than 4 were significantly associated with children’s overall well-being, only DMFT and DT were predictors of negative overall OHRQoL. In previous studies with the same age group, Foster Page et al. [13] found that a DMFS score of 4 or more was associated with a negative impact on overall OHRQoL through oral symptoms and social well-being, while Brown A and Al-Khayal Z [16] found that the DMFT was only significantly correlated with oral symptoms. In our study, the number of carious teeth was significantly associated with oral symptoms and functional limitation, while DMFT was a major predictor of a lower quality of life. All of these studies support the concept that caries experience has a negative impact on quality of life, so the discrepancy is mainly due to how the data are analyzed. Foster Page et al. [13] categorized DMFS scores into four categories (similar to our study), whereas Brown and Al-Khayal [16] evaluated the correlation between the CPQ_11–14_ scores and DMFT. As caries severity can be reflected on the PUFA index, children with a PUFA score of more than 1 were 33 % more likely to be negatively affected. The present study is the only one thus far to investigate the PUFA index and OHRQoL.

In addition to caries, the number of filled teeth could have association with OHRQoL. Children who had 2 or more filled teeth (FT ≥ 2) were less likely to face negative impact experience on overall OHRQoL and especially less likely to develop oral symptoms. Although not a predictor of better quality of life, RI was associated with better quality of life in the oral symptoms domain. Fissure sealants were also predictors of better emotional well-being. There are currently no studies evaluating the effect of fillings or fissure sealants on OHRQoL with which to compare our findings. This indicates that any preventive dental services, such as fissure sealants, may have an effect on the children’s emotional well-being domain of oral health quality of life. Hypodontia has been also proven to negatively affect OHRQoL [30]. Similarly, in our study, the number of missing teeth was a predictor of the emotional distress component of OHRQoL. Interestingly, PI scores were associated with more effects on overall OHRQoL, especially the oral symptoms and functional limitations subscales. Such findings reinforce the fact that healthy oral hygiene practices can have a positive impact on OHRQoL.

This cross-sectional study has inherent limitations due to its cross-sectional design and the use of questionnaires that may have been subject to information bias. Self-administered questionnaires have limitations in identifying cause–effect relationships, but can still show useful associations [31]. Nonetheless, self-reports of oral health-related behaviors and OHRQoL can provide accurate information [32]. Using validated questionnaire and a representative sample may diminish the effects of these limitations. Additional limitation is that malocclusion and dental traumatic injuries were not included in the present study as they may have an impact on children’s OHRQoL. However, caries and plaque level were the main focus of the present study. The number of recruited participants was larger than the calculated sample size as the response rate was higher than the response rate reported previously [19]. All children with positive consents were included to benefit from the clinical examination and the customized oral health advice provided in study.

## Conclusions

In conclusion, our results showed, despite the fact that Kuwaiti school children considered as high caries population, their oral health quality of life was comparable to published reports that evaluated children with lower level of caries. Children’s oral hygiene (plaque level) could be related to overall OHRQoL. Dental caries level, non-cavitated lesions (early lesions), and severe carious lesions may impact oral health symptoms domain; however, RI reflects the treatment received, the higher the RI, the lower the functional limitation subscale scores. Missing and filled teeth were also correlated with the emotional well-being component. Surprisingly, fissure sealants as a dental decay preventive measure positively associated with emotional and well-being component. Further qualitative studies are recommended to evaluate how oral health preventive measures are associated with the emotional and social well-being components of CPQ_11–14_.

## Abbreviations

OH: Oral health
OHS: Oral health status
DHS: Dental health status
OHRQoL: Oral health-related quality of life, DMFT/dmft, Decayed, missed, filled teeth
DMFS/dmfs: Decayed, missed, filled surfaces
RI: Restorative index
PUFA: Pulp, ulcers, fistula, abscess index
PI: Plaque index
CPQ_11–14_: Child’s Perception Questionnaire 11–14
STROBE: Strengthening the reporting of observational studies in epidemiology
WHO: World Health Organization
ICIDAS: International Caries Detection and Assessment System
SD: Standard deviation
OR: odds ratio
ANOVA: Analysis of variance
